# Prediction of lung cancer using novel biomarkers based on microbiome profiling of bronchoalveolar lavage fluid

**DOI:** 10.1038/s41598-024-52296-w

**Published:** 2024-01-19

**Authors:** Gihyeon Kim, Changho Park, Young Kwang Yoon, Dongil Park, Jeong Eun Lee, Dahye Lee, Pureum Sun, Shinyoung Park, Changhee Yun, Da Hyun Kang, Chaeuk Chung

**Affiliations:** 1 Genome and Company, Pangyo‐ro 255, Bundang‐gu, Seongnam, Korea; 2 Department of Internal Medicine, College of Medicine, Chungnam National University, Daejeon, Korea; 3 Institute for Medical Sciences, College of Medicine, Chungnam National University, Daejeon, Korea

**Keywords:** Lung cancer, Diagnostic markers

## Abstract

There is an unmet need for biomarkers for the diagnosis of lung cancer and decision criteria for lung biopsy. We comparatively investigated the lung microbiomes of patients with lung cancer and benign lung diseases. Patients who underwent bronchoscopy at Chungnam National University Hospital between June 2021 and June 2022 were enrolled. Bronchoalveolar lavage fluid (BALF) was collected from 24 patients each with lung cancer and benign lung diseases. The samples were analyzed using 16S rRNA-based metagenomic sequencing. We found that alpha diversity and the beta diversity distribution (*P* = 0.001) differed significantly between patients with benign lung diseases and those with lung cancer. Firmicutes was the most abundant phylum in patients with lung cancer (33.39% ± 17.439), whereas Bacteroidota was the most abundant phylum in patients with benign lung disease (31.132% ± 22.505), respectively. In differential abundance analysis, the most differentially abundant microbiota taxon was unclassified_SAR202_clade, belonging to the phylum Chloroflexi. The established prediction model distinguished patients with benign lung disease from those with lung cancer with a high accuracy (micro area under the curve [AUC] = 0.98 and macro AUC = 0.99). The BALF microbiome may be a novel biomarker for the detection of lung cancer.

## Introduction

Lung cancer is one of the most frequently diagnosed cancers and a prominent cause of cancer-related deaths worldwide^1^. Despite innovations in lung cancer treatment, such as targeted therapy and immunotherapy, many patients experience lung cancer recurrence and progression^2–4^. The early detection of lung cancer is important to improve its long-term prognosis. Low-dose computed tomography (CT) is currently recommended for high-risk individuals^5^. A lung biopsy is essential for the diagnosis and optimal treatment of lung cancer^6^. However, lung biopsy is relatively invasive compared to biopsy of other organs such as the breast and thyroid, and sometimes causes severe complications, including massive hemoptysis and fatal pneumothorax^6^. Therefore, intermediate nodules on CT make it difficult for clinicians to decide whether to perform a lung biopsy or observe for several months. When a lung mass is observed on chest CT, it is often difficult to distinguish between mass-like consolidation caused by pneumonia and lung cancer accompanied by necrosis based on radiological findings alone. Blood biomarkers such as carcinoembryonic antigen and cytokeratin 19 fragments are utilized for the detection of lung cancer^7^. However, they have not yet been fully validated^7,8^. There is still an unmet need for biomarkers for the diagnosis of lung cancer and decision criteria for lung biopsy.

The microbiome is the genetic material of all microorganisms, including bacteria, fungi, protozoa, and viruses, that live in the body^9^. With advances in 16S rRNA gene sequencing, microbiomes in several organs have been actively studied^10^. Many studies have revealed that gut microbiota is associated not only with gastrointestinal disorders but also with systemic diseases, various cancers, and brain diseases^10,11^. Early research focused on the gut microbiota because it contains dense bacterial populations, and many other sites, including the respiratory tract, are thought to be sterile^12^. However, it has recently become clear that the lower respiratory tract also has a dynamic bacterial population that migrates and eliminates the microbiota from the gastrointestinal and upper respiratory tracts^13^. Several studies have investigated the role of the lung microbiome in lung cancers. It has been revealed that an abnormal lung microbiota is associated with the development and progression of pulmonary diseases, including chronic obstructive pulmonary disease, asthma, idiopathic pulmonary fibrosis, and lung cancer^14–18^.

The lung microbiota plays a significant role in regulating mucosal immunity and balancing immune tolerance and inflammation^13^. Several studies have suggested that the lower airway microbiota can affect lung carcinogenesis through various mechanisms, including host inflammation, bacterial toxin production, and the release of cancer-promoting metabolites^19,20^. In lung microbiome studies, researchers have analyzed saliva and sputum specimens or lower respiratory tract samples obtained invasively from bronchoalveolar lavage fluid (BALF) or lung cancer tissue^13^. Because saliva and sputum specimens may have interference from the oral microbiota and lung cancer tissue is often difficult to obtain from patients with advanced lung cancer, BALF is a suitable option for capturing the lung microbiome^21^.

To identify the differences in the lung microbiome between lung cancer and benign lung diseases and to establish a prediction model for lung cancer, we collected BALF from patients with lung cancer and other pulmonary diseases, including pneumonia, bronchiectasis, and interstitial lung disease.

## Methods

### Study population and sample collection

This study enrolled patients who underwent bronchoscopy at the Chungnam National University Hospital between June 2021 and June 2022. BALF was collected from 24 patients with lung cancer and 24 patients with benign lung diseases. BAL was performed on the sides of the lung lesions by a flexible bronchoscopy specialist using a sterile bronchoscope. Three milliliters of BALF were collected from each patient and centrifuged (20,000 relative centrifugal force) at 4 °C for 30 min. One milliliter of DNA/RNA shield was added and the samples were stored at − 80 °C in a microcentrifuge tube. This study adhered to the Declaration of Helsinki and Good Clinical Practice guidelines and was approved by the Institutional Review Board (IRB No. 2021-06-007). Written informed consent was obtained from all patients before participating in this study.

### DNA extraction

DNA was extracted from BALF samples using the Mag-Bind Universal Pathogen Kit (Omega) according to the manufacturer’s protocol. Multiskan GO (Thermo Fisher Scientific) was used to estimate the DNA purity and quantity.

### Bacterial 16S rRNA sequencing of BALF samples

The bacterial 16S rRNA V3–V4 region was amplified using the Illumina 16S Metagenomic Sequencing Library Preparation guide (Illumina) and primers with adapter overhang sequences^22^. Forward primer: 5′-TCGTCGGCAGCGTCAGATGTGTATAAGAGACAGCCTACGGGNGGCWGCAG-3′, reverse primer: 5′-GTCTCGTGGGCTCGGAGATGTGTATAAGAGACAGGACTACHVGGGTATCTAATCC-3′. The 25-µL PCR mixture contained 2 µL of genomic DNA, 0.5 µL of each primer, 12.5 µL of 2 × KAPA HiFi HotStart ReadyMix (Kapa Biosystems), and 9.5 µL of distilled water. The PCR conditions were as follows: 95 °C for 3 min for pre-denaturation of the DNA; 25 cycles at 95 °C for 30 s for denaturation, 50 °C for 30 s for annealing, and 72 °C for 30 s for extension; and 72 °C for 5 min for the final extension. The PCR products were purified using AMPure XP Beads (Beckman Coulter). Dual index adapters and Illumina sequencing adapters were added using PCR products (5 µL), Illumina Nextera XT Index Primer 1 (5 µL, N7xx), Nextera XT Index Primer 2 (5 µL, S5xx), 2 × KAPA HiFi HotStart Ready Mix (25 µL), and nuclease-free water (10 µL) using the following thermal cycles: 95 °C for 3 min; 8 cycles of 95 °C for 30 s, 55 °C for 30 s, and 72 °C for 30 s; and 72 °C for 5 min. The PCR products were cleaned using AMPure XP beads and quality control of the 16S metagenomic libraries was performed using an Agilent Technologies 2100 Bioanalyzer (Agilent). Libraries were standardized and pooled for sequencing on a MiSeq platform (Illumina, San Diego, CA, USA) according to the standard Illumina sequencing protocol.

### Metagenomic analysis

The Illumina adapter sequences of the paired-end reads were removed using Cutadapt version 2.2^23^. Trimmed sequences were processed using QIIME2 version 2022.8. Briefly, reads were assigned to each sample according to a unique index, and pairs of reads from the original DNA fragments were merged using an import tool in QIIME2^24^. Quality control and trimming were performed to yield sequences with lengths of 270 and 210 bp for the forward and reverse reads, respectively. The DADA2 software package^25^ in QIIME2 was used to remove low-quality bases from the reads. A consensus method implemented in DADA2 was used to remove chimeras from the FASTQ files. Amplicon sequence variants (ASVs) were filtered out if they were confirmed as contaminants by *decontam*^26^ using the DNA concentration. Only 47 ASVs were filtered out of the total 5092 ASVs (Supplementary File 1). Alpha and beta diversities were calculated using the alpha- and beta-group significance in the QIIME2 diversity plugin and analyzed using core-metrics-phylogenetic analysis in the QIIME2 diversity plugin. Alpha diversity was calculated using the observed features, and beta diversity was compared using principal coordinate analysis with Bray–Curtis distances. The significance of the similarity between groups was evaluated using permutational multivariate analysis of variance (PERMANOVA) with 999 permutations. Taxonomic annotation was performed by mapping the training reference set with primers (forward, 5′-CCTACGGGNGGCWGCAG-3′; reverse, 5′-GACTACHVGGGTATCTAATCC-3′) and extracting the V3–V4 region using Silva (version 138.1). A prediction model was established using the random forest function of the QIIME2 plugin. The following variables were used: number of estimators = 100, random state = 1234, test set size = 0.3, and cross-validation = 10.

### Statistical analysis

Demographic and clinical variables were compared using independent *t*-tests for continuous variables and chi-squared tests for categorical variables. Statistical analyses were performed using GraphPad software (version 9.4.1; Prism, La Jolla, CA, USA). Differences between the two variables were analyzed using the Wilcoxon–Mann–Whitney test for nonparametric values. Statistical significance was set at *P* < 0.05. The detailed statistical methods are described in the figure legends.

### Ethics approval

This study was conducted per the Declaration of Helsinki and approved by the Institutional Review Board of Chungnam National University Hospital (IRB No. 2021-06-007). Informed consent was obtained from all participants involved in the study.

## Results

### Clinical characteristics of the study population

We enrolled 48 patients between June 2021 and June 2022. A total of 24 patients were pathologically diagnosed with lung cancer, and 24 were diagnosed with benign lung diseases, including pneumonia, anthracofibrosis, bronchitis, and bronchiectasis. Table 1 presents the baseline characteristics of the two groups. The mean age of the patients was 66.2 years (range, 45–81 years), with a preponderance of male (77.1%) patients. The mean body mass index (BMI) was 21.86 kg/m^2^ (range, 14.22–27.93). There were no significant differences in age, BMI, sex, and smoking status between the two groups. Histological subtypes among lung cancer patients were adenocarcinoma in 29.2%, squamous cell carcinoma in 54.2%, and small cell carcinoma in 16.7%. All patients were diagnosed with stage III or IV lung cancer. A total of 29.2% (7/24) of patients had high PD-L1 expression, and 58.3% (14/24) had no or low PD-L1 expression. A majority of the benign lung disease group comprised patients with pneumonia (45.8%). We defined pneumonia based on the clinical and radiographic findings of the pulmonologists. All patients with pneumonia included in this study were community-acquired pneumonia cases and treated in outpatient settings with low severity, with a CURB-65 score of 0 or 1. This study mainly included patients with pneumonia who required bronchoscopy because they showed mass-like consolidation that needed to be differentiated from malignancy on radiological examination. Only patients without previous exposure to broad-spectrum antibiotics and glucocorticoids were included.

**Table 1:** Baseline characteristics of patients (N = 48).

Variable	Patients with lung cancer (N = 24)	Patients with benign lung diseases (N = 24)	*P*-value
Age, years	67.7 ± 9.7	64.7 ± 9.4	0.276
BMI	21.6 ± 3.6	22.3 ± 4.8	0.547
Sex			
Male	20 (83.3)	17 (70.8)	0.494
Female	4 (16.7)	7 (29.2)	
Smoking status			
Never	5 (20.8)	11 (45.8)	0.125
Former/current	19 (79.2)	13 (54.2)	
Histology			
Adenocarcinoma	7 (29.2)		
Squamous	13 (54.2)		
Small cell carcinoma	4 (16.7)		
EGFR			
Mutant	2 (8.3)		
Wild type	22 (91.7)		
PD-L1 expression†			
Unknown	3 (12.5)		
No/low (TPS 0–49%)	14 (58.3)		
High (TPS ≥ 50%)	7 (29.2)		
Stage			
III	10 (41.7)		
IV	14 (58.3)		
Benign lung disease			
Pneumonia		11 (45.8)	
Lung nodule		1 (4.2)	
Anthracofibrosis		2 (8.3)	
Bronchiectasis		3 (12.5)	
Bronchitis		2 (8.3)	
Interstitial lung disease		1 (4.2)	
Others		4 (16.7)	

*EGFR* epidermal growth factor receptor, *PD-L1* programmed death-ligand 1, *TPS* tumor proportion score.

^†^The classification of subgroups according to PD-L1 expression was based on the results of the 22C3 pharmDx assay, and patients without 22C3 pharmDx assay results were classified based on the SP263 assay.

### The BALF microbiome in lung cancer shows high alpha diversity

**Figure 1:**
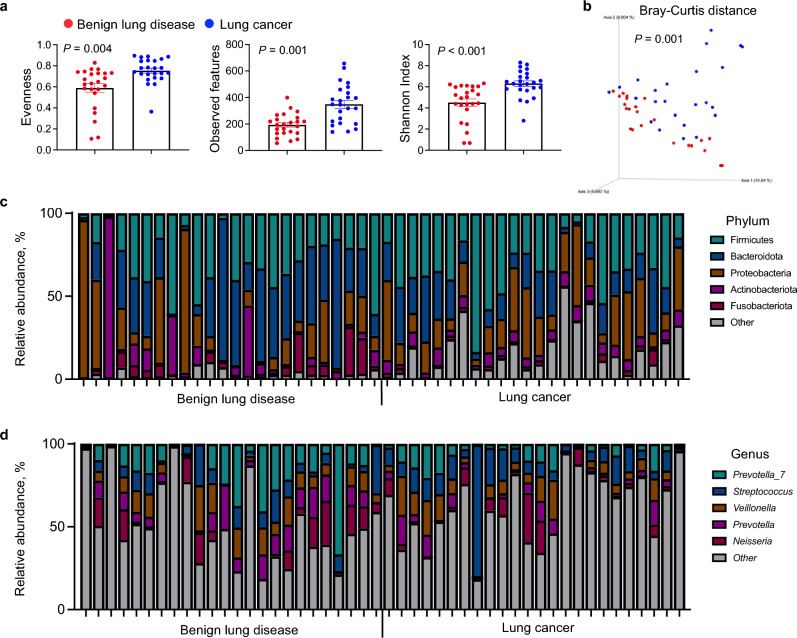
Microbial diversity and relative abundance in benign lung disease and lung cancer patients. (**a**) Comparison of alpha diversity between benign lung disease patients and lung cancer patients. Evenness, observed features, and Shannon index were calculated. (**b**) Beta diversity (Bray–Curtis distance) in benign lung disease and lung cancer patients. The statistical significance of differences in alpha and beta diversity was calculated using the Wilcoxon–Mann–Whitney test and PERMANOVA with 999 permutations, respectively. Error bars represent the distribution of diversity scores. Numbers in graphs indicate *P*-values representing the difference of alpha and beta diversities between groups. Color corresponds to the group. Taxonomic profiles at the phylum level (**c**) and genus level (**d**) in benign lung disease and lung cancer patients. N = 24 per group.

To assess the potential association of the lung microbiome with lung cancer, we performed 16S rRNA sequencing of BALF from benign lung disease patients and lung cancer patients. We identified significantly different alpha diversities between patients with benign lung diseases and those with lung cancer (Fig. 1a). Evenness (*P* = 0.004), observed features (*P* = 0.001), and the Shannon index (*P* < 0.001) were higher in the BALF samples of patients with lung cancer compared to those of patients with benign lung diseases. We also observed a significant difference in the beta diversity distribution (*P* = 0.001) (Fig. 1b).

### Differences in the BALF microbiome profiles of lung cancer and other pulmonary diseases

We profiled taxonomic information at the phylum and genus levels. Firmicutes was the most abundant phylum in patients with lung cancer (33.39% ± 17.439), whereas Bacteroidota was the most abundant phylum in those with benign lung diseases (31.132% ± 22.505) (Fig. 1c). The ratio of Firmicutes to Bacteroidetes was significantly higher in patients with lung cancer than in those with benign lung diseases (*P* = 0.005) (Supplementary Fig. 1). Proteobacteria, Actinobacteria, and Fusobacteria were detected in patients with benign lung diseases and lung cancer (Fig. 1c). At the genus level, *Prevotella_7* was the most abundant in patients with benign lung diseases (15.068% ± 15.76), whereas *Streptococcus* was the most abundant in patients with lung cancer (12.67% ± 15.245) (Fig. 1d). Interestingly, *Streptococcus* was the most important bacterial species responsible for beta diversity in patients with benign lung diseases and lung cancer (Supplementary Fig. 2a). We also found that the *Streptococcus* levels were significantly higher in patients with lung cancer (*P* = 0.12) (Supplementary Fig. 2b). Pneumonia is closely associated with the lung microbiota and is the most common disease among patients with benign lung diseases. Therefore, we compared the microbial communities in patients with pneumonia and lung cancer. Interestingly, we observed lowest alpha diversity in BALF samples from patients with pneumonia compared to those with lung cancer and other benign lung diseases, consistent with a previous study^27^ (*P* = 0.0001) (Supplementary Fig. 2c). The microbial communities were significantly different (*P* = 0.001) and *Streptococcus* was significantly related to this microbial distribution (Supplementary Fig. 2d). However, we did not observe any significant differences in the microbial communities among patients with lung cancer (Supplementary Fig. 3).

### The SAR202 clade of the phylum Chloroflexi is abundant in lung cancer

**Figure 2:**
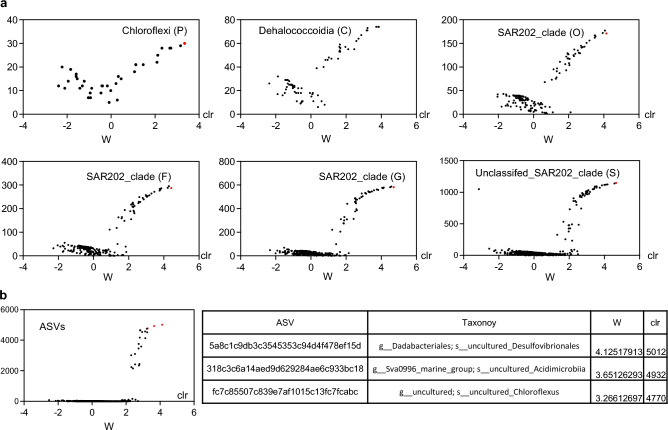
Differentially abundant microbiota taxa between patients with benign lung disease and lung cancer. The differentially abundant microbiota taxa were analyzed (**a**) at specific taxonomic levels (phylum, class, order, family, genus, and species levels) and (**b**) amplicon sequence variants were filtered. The X-axis indicates the center log-ratio and the Y-axis indicates W statistics. The most differentially abundant microbiota taxon is indicated as a red dot.

To investigate the specific microbiota taxa associated with lung cancer, we performed a differential abundance analysis of the microbiota using an analysis of composition of microbiomes (ANCOM)^28^. We compared microbiota abundance at various taxonomic levels and found that most of the microbiota taxa were more abundant in patients with lung cancer than in those with benign lung diseases (Fig. 2a). The most distinct microbiota taxon was unclassified_SAR202_clade, belonging to the phylum Chloroflexi (Fig. 2a). *Chloroflexus*, Sva0996_marine group, and Dadabacteriales were abundant in patients with lung cancer (Supplementary file 2). Consistent herewith, we observed that amplicon sequence variants identified as *Chloroflexus*, Sva0996_marine group, and Dadabacteriales were highly abundant in patients with lung cancer (Fig. 2b). The SAR202 cluster, belonging to the phylum Chloroflexi, is the first microbial lineage discovered to specifically inhabit the aphotic realm, where it is abundant and globally distributed^29^. The same result was observed using linear discriminant analysis effect size (LEfSe) analysis (Supplementary Fig. 4)^30^.

### Prediction of lung cancer using a random forest model

**Figure 3:**
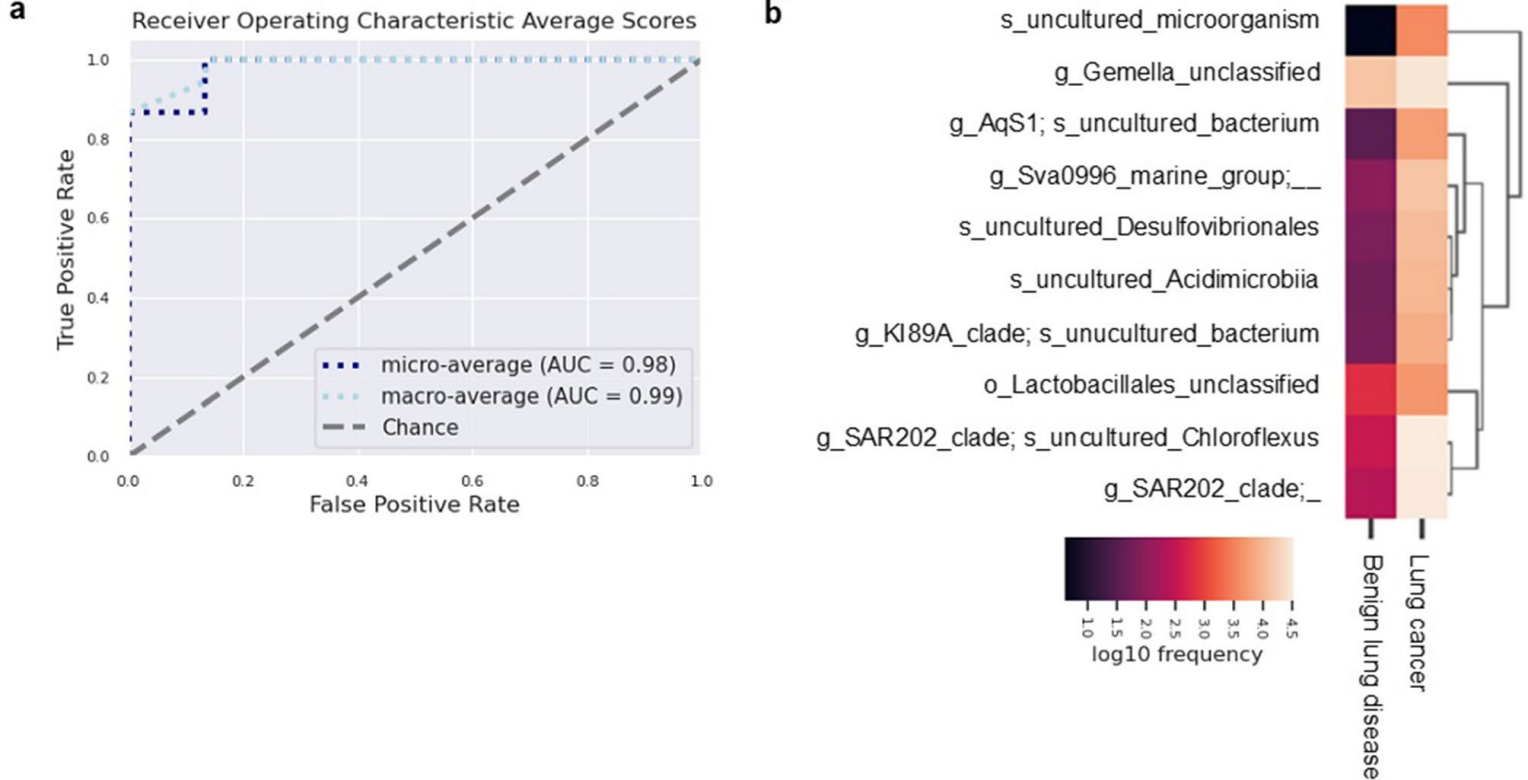
Prediction model for benign lung diseases and lung cancer. (**a**) Receiver operating characteristic curve for the prediction model. AUCs for micro-average and macro-average are indicated. (**b**) Frequency of the top 10 most important taxa in the prediction model.

As the microbial community and composition were significantly different between patients with benign lung diseases and those with lung cancer, we established a prediction model for lung cancer based on the BALF microbiome using a random forest model. We ran the training with a subgroup of 33 patients and tested the model in the remaining 15 patients with 10 cross-validations (details in Methods). The prediction model distinguished patients with benign lung diseases from those with lung cancer with high accuracy (micro area under the curve [AUC] = 0.98, macro AUC = 0.99) (Fig. 3a). Important taxa were SAR202_clade (uncultured bacterium, uncultured Chloroflexi, and uncultured *Chloroflexus*) and uncultured *Acidobacterium*, and these taxa were more abundant in lung cancer patients than in benign lung disease patients (Fig. 3b). Additionally, we established a predictive model for patients with pneumonia and lung cancer. The prediction model distinguished between patients with pneumonia and lung cancer (micro AUC = 0.94, macro AUC = 0.98) (Supplementary Fig. 5a). The SAR202_clade, *Chloroflexus* (uncultured), *Neissera* (unclassified), and *Veillonella* (unclassified) were important taxa in this prediction model (Supplementary Fig. 5b).

## Discussion

In this study, we investigated the composition of the lung microbiota in BALF from patients with lung cancer and benign lung diseases. This study revealed that the lung microbiota diversity in lung cancer patients was higher than that in patients with benign lung diseases; Firmicutes was the most abundant phylum in lung cancer patients, whereas Bacteroidetes was the most abundant phylum in patients with benign lung diseases. In addition, the SAR202 clade of the phylum Chloroflexi was significantly more abundant in patients with lung cancer. We established a predictive model based on this finding.

Clinical characteristics, including age, sex, BMI, and smoking status, can affect bacterial communities^31,32^. Therefore, to ensure the reliability of the analysis, we first confirmed that there were no significant differences in these factors between the two groups. In the alpha-diversity analysis, evenness, observed features, and Shannon index were significantly higher in lung cancer patients than in benign lung disease patients, which is similar to previous findings^18,33,34^. In contrast, several reports described no significant difference in the richness and alpha diversity of the microbiota between patients with lung cancer and controls^21,35,36^. We believe that the differences in these results may be due to various factors, including the diversity of diseases in the non-cancer groups in each study, environmental factors, air exposure, patient diet, and the depth of microbiota sequencing.

Our study showed that the relative abundance of Firmicutes was significantly elevated in the BALF samples from patients with lung cancer, and the ratio of Firmicutes to Bacteroidetes was significantly higher in patients with lung cancer than in those with benign lung diseases. Firmicutes and Bacteroidetes are the two phyla that are mainly present in healthy lungs^37,38^. Several studies have reported a higher prevalence of Firmicutes in patients with lung cancer than in controls^34,39^. A previous study has shown that the proportion of Firmicutes is significantly higher in lung cancer patients than in patients with benign lesions, especially in lung cancer patients with a smoking history, where a significantly higher Firmicutes to Bacteroidetes ratio was observed, suggesting that Firmicutes may be associated with smoking^18^. In our study, although more smokers were included in the lung cancer group than in the benign lung disease group, the difference was not statistically significant. This suggests that the abundance of Firmicutes may not only be due to smoking but may also be a result of lung cancer. In addition, *Streptococcus* was the most abundant genus in patients with lung cancer. In previous studies using saliva and sputum, the proportion of *Streptococcus* was lower in patients with lung cancer than in controls^19^. However, several studies have reported a higher prevalence of *Streptococcus* in cancer patients than in controls using samples obtained from lung tissue or bronchial brushing^34,40,41^. We obtained similar results in lung tissues using BALF samples, demonstrating that BALF samples can also represent the microbial community status of the lungs. Based on the microbial analysis results in this study, *Streptococcus* may influence the development of lung cancer. A recent study has reported that *Streptococcus pneumoniae* promotes lung cancer development and progression^42^.

In the differential abundance analysis, unclassified_SAR202_clade belonging to the phylum Chloroflexi was the most differentially abundant taxon between patients with lung cancer and those with benign lung diseases. A study comparing the microbiomes of lung adenocarcinoma tumor tissue and paired adjacent normal tissue reported a significant difference in the phylum Chloroflexi in sub-solid nodules compared to solid nodules^43^. However, they did not find a significant difference in the proportion of Chloroflexi between normal and tumor tissues, and no significant difference was observed at the genus or strain level in the bacteria belonging to this phylum. Our study is the first to demonstrate a significant difference in SAR202_clade, belonging to Chloroflexi, between lung cancer and benign lung diseases. SAR202_clade was first discovered in seawater during an early investigation of bacterioplankton in the North Atlantic Ocean^44^. SAR202 species are the most abundant lineage of bacteria in deep oceans^45^. Notably, the results of this study may be affected by the region where patients live and differences in their diets. However, our institution is located in the city of Daejeon, which is at the center of South Korea and is not close to the sea. Almost all patients visiting our institution were from Daejeon or rural areas near Daejeon far from the sea. Additionally, no specific differences in diet were found between patients with lung cancer and those with benign lung diseases. To the best of our knowledge, SAR202 species have not previously been detected or reported in BALF samples from patients with lung cancer. Based on these results, a lung cancer prediction model was developed that showed very high predictive accuracy, with an AUC of 0.85–0.93. In this study, the proportion of cancer diagnosed through bronchoscopic biopsy was 83.3% (20/24), and atypical cells were confirmed in two patients (8.3%). Two cases (8.3%) had insufficient results, such as anthracosis and non-neoplastic epithelium. In cytology analysis using the same BAL samples as in the microbiome analysis of this study, malignant cells were positive in 5 out of 24 patients, atypical cells were confirmed in 3 patients, and malignant cells were negative in 16 patients. Thus, the sensitivity of BAL cytology test was only 33.3% even including atypical cells. Even considering that cytology tests have high specificity, there is a problem with their sensitivity being too low. The prediction model based on microbiome composition showed high sensitivity and specificity Therefore, although it is difficult for this prediction model to replace biopsy, it is expected to be able to predict lung cancer much better than BAL cytology. SAR202_clade is particularly important for distinguishing between lung cancer and benign lung disease in this prediction model. Further research is needed to investigate why SAR202_clade belonging to Chloroflexi is abundant in lung cancer patients and why a microbiota taxon originating from the ocean is present in BALF samples from the human respiratory tract.

Our study has some limitations which warrant further consideration. First, the number of patients enrolled in this study was not sufficient to analyze and classify the histological types, stages, and treatment responses in patients with lung cancer. Second, the types of benign lung diseases included in this study were diverse, and validation was not conducted. It is very important to perform validation of the prediction model in an independent patient cohort. For validation, we are collecting additional BAL samples from patients with benign lung disease and lung cancer prospectively. We plan to conduct microbiome analysis in the same protocol in the future. Further large-scale studies are required to validate our results and explore the role of the microbiota in patients with lung cancer. Third, low biomass samples in intricate DNA solution could be removed through QC. To address this limitation, performing a digital droplet PCR is suitable for detecting low biomass in a future study. Fourth, although we avoided suction until the endoscope approached the lesion site to minimize upper airway contamination during the procedure, the possibility of upper airway contamination cannot be completely excluded. In addition, since the SAR202 clade of the phylum Chloroflexi is known to originate from ocean waters, there may be other potential factors that may affect the results of this study other than patients’ residential areas or diets. More investigation and research are needed in these areas in the future.

## Conclusions

We found considerable differences in BALF microbiome profiles of lung cancer and other pulmonary diseases. We identified, for the first time, that the SAR202 clade of the phylum Chloroflexi is distinctively abundant in lung cancer. Machine learning prediction using BALF microbiome characteristics significantly differentiated lung cancer from benign diseases. We conclude that the BALF microbiome may be a novel biomarker for the detection of lung cancer.

### Supplementary Information

Supplementary Figures.

Supplementary Information 1.

Supplementary Information 2.

## Data Availability

The data presented in this study are available upon request from the corresponding author. The data are not publicly available because of patient privacy concerns.
