# Supplementary material for: Prediction of lung cancer using novel biomarkers based on microbiome profiling of bronchoalveolar lavage fluid
Source: Sci Rep. 2024 Jan 19;14:1691. doi: 10.1038/s41598-024-52296-w (PMC10799071; doi:10.1038/s41598-024-52296-w)
Supplement: Supplementary file 1 — Supplementary Figures. [file 41598_2024_52296_MOESM1_ESM.docx]

**Supplementary Figure 1. Ratio of Firmicutes and Bacteroidota in BAL from patients with benign lung disease and those with lung cancer.** Ratio was calculated by using relative abundance of each phylum. Error bars represent the distribution of the ratio. Numbers in graphs indicate P-values representing the difference of ratio. Statistical significance was calculated by using Wilcoxon-Mann-Whitney test. Color corresponds to group.

**Supplementary Figure 2. Beta diversity using the biplot. (a)** Biplot of Bray-Curtis distance of patients with benign lung disease and lung cancer. Enlarged arrow of biplot is shown in the subplot. **(b)** Dotplot showing the relative abundance of Streptococcus in patients with benign lung disease and lung cancer; n = 24 per group. Statistical significance of difference was calculated by Wilcoxon-Mann-Whitney test. **(c)** Bray-Curtis distance of patients with pneumonia (n = 11) and lung cancer (n = 24). **(d)** Biplot of Bray-Curtis distance of patients with pneumonia and lung cancer. Enlarged arrow of biplot is shown in the subplot. Statistical significance of beta diversity is calculated using PERMANOVA with 999 permutations.

**Supplementary Figure 3. Beta diversity of patients with lung cancer by subtype.** Statistical significance of beta diversity was calculated by PERMANOVA with 999 permutations. Color corresponds to the subtype of lung cancer. *n =* 24. Cytotoxic Chemo, cytotoxic chemotherapy. FU_loss, follow up loss. Supportive, supportive care. Targeted, targeted therapy. IO-chemo combi, combinations of immune checkpoint inhibitor and cytotoxic chemotherapy.

Supplementary Figure 4. Bar graph shows the result of LEfSe analysis between patients with benign lung diseases and lung cancer. Statistical significance was calculated by Wilcoxon-Mann-Whitney test.

**Supplementary Figure 5. Prediction model for patients with pneumonia and lung cancer. (a)** Receive Operating Characteristic curve for prediction model. Area under the curve (AUC) for micro-average and macro-average. **(b)** Frequency of the top 10 important microbiomes in the prediction model.
